# Supplementary material for: Endocannabinoid System Receptors at the Hip and Stifle Joints of Middle-Aged Dogs: A Novel Target for the Therapeutic Use of Cannabis sativa Extract in Canine Arthropathies
Source: Animals (Basel). 2023 Sep 6;13(18):2833. doi: 10.3390/ani13182833 (PMC10525782; doi:10.3390/ani13182833)
Supplement: Supplementary file 1 [file animals-13-02833-s001.zip › Figure S1.pdf]

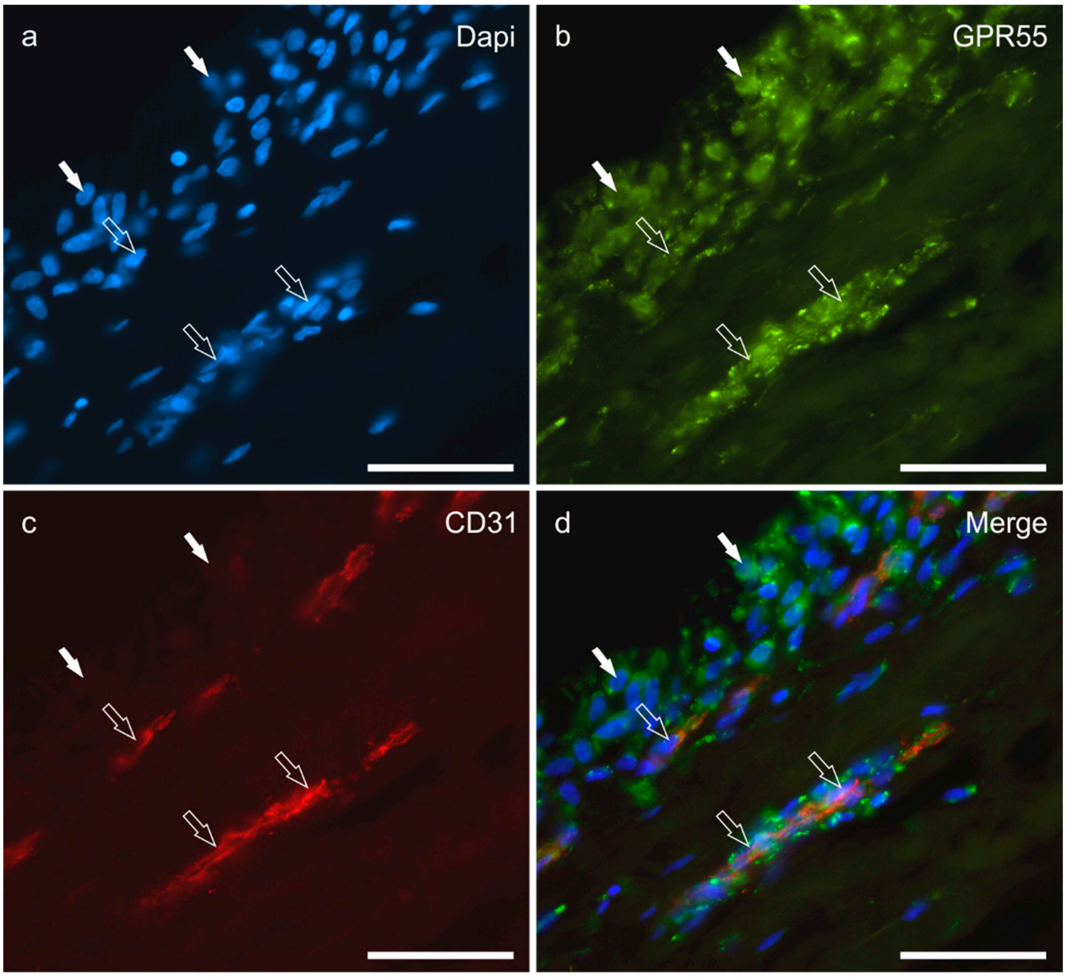

~~**Figure S4.**~~**Figure S1.** Photomicrographs of cryosections of the synovial membrane of the stifle joints of dogs showing immunoreactivity for the G Protein-Coupled Receptor 55 (GPR55) (b) and for the endothelial marker CD31 (c). The white arrows indicate synoviocytes expressing bright GPR55 immunoreactivity (b). The open arrows indicate the Dapi labelled nuclei of the endothelial cells of the subintimal capillaries co-expressing CD31 and GPR55 immunoreactivity. Bar: 50 μ
